# Translation and validation of the revised and short forms of the Death Literacy Index in the Chinese population

**DOI:** 10.3389/fpubh.2026.1701996

**Published:** 2026-01-30

**Authors:** Wai I Ng, Sok Leng Che, Sio Leng Wong, Meng Fa Wong, Wai Tan Tammy Yung, Ka Meng Ao, Qun Wang, Jie Pan

**Affiliations:** 1 Kiang Wu Nursing College of Macau, Macao, Macao SAR, China; 2 Kiang Wu Hospital, Macao, Macao SAR, China; 3 School of Nursing, Shenzhen University, Shenzhen, Guangdong, China; 4 Department of Nursing, School of Medicine, Foshan University, Foshan, Guangdong, China

**Keywords:** Chinese, death literacy, Death Literacy Index, public palliative care, scale validation

## Abstract

**Introduction:**

Death literacy has gained attention in recent years, and the use of the Death Literacy Index (DLI) has been increasing. It measures knowledge about the death system. After being translated and used in multiple countries, the original authors incorporated feedback from various countries and revised the DLI to become the DLI-R. A shorter version, the DLI-9, was also developed for practical use. This study aimed to validate the DLI-R and DLI-9 in the Chinese population.

**Methods:**

The DLI was forward- and backward-translated into Chinese by two expert panels. A pilot test was conducted before the main survey. A total of 1,147 participants were recruited online from three cities in southern China (Shenzhen, Foshan, and Macao) to examine the factor structure, validity, and reliability of the translated DLI-R and DLI-9.

**Results:**

Exploratory factor analysis showed a five-factor structure. The Cronbach’s alpha of the Chinese DLI-R was 0.92, and the five factors were between 0.78 and 0.95, accounting for 65.06% of cumulative variance. The Cronbach’s alpha of the Chinese DLI-9 was 0.79, accounting for 52.56% of cumulative variance. The five-factor structure was confirmed by a confirmatory factor analysis. The overall scale and subscales showed high internal consistency reliability and satisfactory validity.

**Discussion:**

The Chinese DLI-R was shown to be a reliable and valid instrument for measuring death literacy among individuals in southern China and is suitable for both research and clinical use. Several demographic characteristics, cultural adaptation issues, and applicability considerations were also identified for the Chinese DLI-9.

## 1 Background

Death literacy is a concept introduced by Horsfall and the Caring at the End of Life Research Team of Australia in 2020, developed through a series of studies investigating the social and caring resources and support in end-of-life care (1). Death literacy is defined as the knowledge and skills that enable individuals to access resources and make end-of-life decisions and death care choices (1). Individuals with higher levels of death literacy are believed to be more capable of accessing, understanding, and utilizing end-of-life caring services, policies, and healthcare procedures, as well as engaging in end-of-life planning (2).

The Death Literacy Index (DLI) was developed accordingly by the Australian team, and it is a population-based measure that was constructed to examine the quality of the healthcare interventions at the end-of-life (3). It aims to be used by community palliative care practitioners and researchers for assessing intervention effects on individuals, communities, societies, or even a nation at large (1). Multiple international studies have translated and validated DLI in different cultural settings. Che’s (4) team has translated DLI into traditional Chinese and simplified Chinese, which enabled adaptation and applicability to various contexts of Chinese culture. DLI was also translated into Swedish (5), Turkish (6), Flemish, and Dutch (7) and validated with the overall scale and subscales, demonstrating good structural and construct validity and internal consistency reliability. Despite the Swedish version reporting variance in measuring community capacity subscales compared to other subscales in the Swedish context, Johansson et al.’s (7) cross-national study demonstrated configural, scalar, and metric invariance in a series of multigroup confirmatory factor analyses of DLI across populations of several countries (Belgium, the Netherlands, and Sweden) speaking different languages, and it was supported to be used in cross-national studies in comparing and evaluating the effects and effectiveness of community intervention initiatives. In the UK, the DLI was used to investigate the death literacy level of the population and performed a population-level benchmark (8). In China, a wide survey with 2,002 participants was conducted to understand the level of death literacy of the Chinese population in the Guangdong-Hong Kong-Macao Greater Bay Area and propose a responsive life and death education system for the region (9).

As the DLI has been gaining international attention, the original research team revised the scale (DLI-R) and developed a short form (DLI-9) to respond to the concerns raised in wording and readability of the original DLI, and that may potentially affect the willingness to apply the tool in different cultural contexts (10). Upon the requests of community and research groups, a short form with 9 items (DLI-9) was also developed to address the demand and to promote a satisfactory response rate and a valid response during the survey. To revise the DLI, the international literature was reviewed. Among 29 items, the wording of 24 items in the DLI was revised for clarity. It showed acceptable psychometric properties; higher interrater reliability between the DLI-R and DLI-9 (ICC = 0.98), high internal consistency reliability in the overall DLI-R and its subscales, and DLI-9 with their Cronbach’s alpha coefficients all above 0.8.

Chinese scholars noted that death literacy presents a new public health perspective on hospice and palliative care in China, and it could support communities to prepare for, deal with, engage in, decide on, and utilize end-of-life care (11). “Healthy China 2023” is a strategic plan issued by the State Council of the People’s Republic of China in 2016. One of its goals was to integrate palliative care into the healthcare and elder care system of China (12). By 2022, 4,259 medical or health institutes across the country had been providing palliative and hospice care. Community health facilities are expected to provide community and home-based palliative and hospice care by 2025 (13). In response to this national development plan, research on death literacy in China has also been steadily increasing. It started with cross-sectional surveys investigating the level of death literacy in different populations (14–16), while an interventional study for evaluating the effectiveness of a death literacy training program is in progress (17). To this end, a rigorous, deliberately validated, and culturally adapted tool is important and anticipated for measuring death literacy in this Chinese context.

Although the DLI has been reported as a valid and reliable tool when it was translated and validated in the southern Chinese population, due to variance by gender and profession, low loadings were observed on several items of the scale (4). Moreover, the DLI-9 is a newly developed short form of the DLI, intended for use in practical community settings (10). However, it is important to note that DLI-9 was neither developed based on rigorous qualitative research nor with a theoretical base, as was done for the DLI. Further research is needed to examine its performance as an engagement tool in community research across diverse cultural contexts. Therefore, this study aimed to validate the DLI-R and DLI-9, providing a promising tool to support the development of public palliative care initiatives among the Chinese population.

## 2 Methods

### 2.1 Study design

A cross-sectional online survey was conducted in three cities of southern China, namely Macao, Foshan, and Shenzhen, to validate the Chinese versions of the DLI-R and DLI-9.

### 2.2 Translation of the DLI-R

The DLI-R was translated in accordance with Tsang’s guidelines (18), which include the establishment of an expert committee, forward translation, backward translation, and pilot testing. The expert committee consists of three nursing PhDs, one English–Chinese translation professional, and two research team members (WIN and SLC), all of whom are fluent in Cantonese, Mandarin, and English. Two experts performed forward translation, while the other two experts performed backward translation.

Forward translation was performed individually. The two experts translated the DLI-R into plain language in both Cantonese and Mandarin Chinese. Cantonese is the official spoken Chinese in both Macao and Hong Kong, and their written Chinese is Traditional Chinese, while Mandarin is the official spoken Chinese in the Mainland, and its written Chinese is Simplified Chinese; both traditional and simplified Chinese versions were translated in this study. After forward translation, a panel consisting of two experts and two research team members reviewed the two translations, discussed the differences between the two translations and the reasons for the differences, and finally confirmed the consistency of the translation.

Considering that DLI-R and DLI-9 have neither replacement of items nor changes in the number of items of DLI, the construct and stem of the items of the tool remain unchanged. Since it was our research team that translated and performed cultural adaptation of DLI into a Chinese version in 2022, instead of simply repeating the cognitive interviews, cultural consideration and applicability were discussed and referred to throughout the review process of DLI-R and DLI-9. For example, in the subscale “Providing hands-on care,” Item 6 “Wash a person” replaced “Bathing a person.” In Chinese, “to bath” is expressed in a different way from “to wash,” so the panel decided to keep the expression of “to bath” to keep the original meaning of DLI-R. In the subscale “Experiential knowledge,” Item 11 “Developed my wisdom and understanding,” the two translators translated the term “understanding” into different expressions, bearing the meaning of “enlightenment” and “cognition” in Chinese. Neither of them could reach a consensus on a Chinese term that best fits the meaning of “understanding” in this item. During the discussion, the Chinese version of DLI was made reference, and the panel agreed to retain the original translation, which means “a perspective gained from experience”; taken together, the consideration of wording of item 11 has not changed in DLI-R. After the panel finalized the Chinese translation, two other translators with similar language abilities to the previous two translators were invited to perform backward translation. A panel discussion was again conducted to reach a consensus on the translation of all items. A change was made to item 20 from “cemetery staff” to “cemetery/funeral staff” in recognition that funeral staff are responsible for managing the entire funeral process, whereas cemetery staff are only one part of that process.

After translation, a pilot test was carried out to examine the clarity and reliability of the translated DLI-R and to identify ambiguity in the translation. In total, 26 participants aged 25–61 (40.62 ± 10.65) were purposively recruited to evaluate the time required to complete the questionnaire, and to identify potential problems. The online questionnaire was sent to the potential participants, with the same question structure as the survey. Participants were asked to identify potential ambiguities in the items and provide feedback after completion of the questionnaire. Verbal feedback revealed that most of the participants were satisfied with the comprehension. The time used was 1796.65 ± 2489.41 s (275–9,713 s). The wording of the items was evaluated using Cronbach’s alpha. The overall Cronbach’s alpha was 0.86. Therefore, there was no change to the items after the pilot test.

### 2.3 Participant recruitment

Participant recruitment took place from January to May 2025 in Macao, Foshan, and Shenzhen. Data collection was conducted through an online survey. Inclusion criteria of the study participants included people living in these three cities during data collection, bearing Chinese ethnicity, aged 18–74, who understood written Chinese and were able to read the study information and provide consent to participate in the study. A sample size of 196 was calculated using the equation for cross-sectional surveys (19). Two e-posters were designed in traditional Chinese or simplified Chinese, stating a brief description of the study and a QR code that can lead to the online questionnaire once scanned. Convenience and snowball sampling were utilized to recruit participants in the three cities through various social media, such as Facebook, WhatsApp, WeChat, and the institute website, where the e-poster was disseminated, and wide forwarding of the e-poster was encouraged. After reading the study description, participants click the consent button to proceed to the questionnaire. In addition, one-to-one interviews were conducted with older adults to assist them in responding to the survey by trained investigators at three community centers.

### 2.4 Questionnaire

The questionnaire consisted of three parts. The first part collected the sociodemographic characteristics of participants, including age, gender, city of residence, level of education, marital status, number of children, religious belief, occupation, income, self-reported health status, any chronic disease, and hospital admission in the past year.

The second part consisted of the Chinese DLI-R, which was the final version of the above translation process. As in the original DLI-R, Chinese DLI-R also contains 29 items constructing four domains, namely “Practical knowledge,” “Experiential knowledge,” Factual knowledge,” and “Community knowledge.” Response options for each item are on a five-point Likert scale. There is no reverse-coded item. Scores were calculated based on the sum of the items and scaled by the number of items in a subscale (with a range between 0 and 10); a higher score indicates higher death literacy.

The third part comprised the Bugen’s Coping with Death Scale (CDS), which is used to examine the convergent validity of the Chinese DLI-R. CDS consists of eight dimensions and 30 items in total. The eight dimensions include “Death acceptance,” “Death processing,” “Death thinking and expression,” “Funeral processing,” “Life inspection,” “Loss processing,” “Ability to discuss others’ deaths,” and “Ability to discuss death” (20). It was translated into traditional Chinese by Tsang (21). It is measured with a six-point Likert scale with 1 representing “Very unsatisfactory” and 6 representing “Very satisfactory.” The total score of the CDS ranges from 30 to 180, and a higher score indicates a higher ability to manage death. The Cronbach’s alpha of CDS was 0.95 in this study.

### 2.5 Statistical analysis and scale evaluation

Raw data were coded using Microsoft 365 Excel, confirmatory factor analysis (CFA) was performed using Amos (version 22.0), and Statistical Package for the Social Sciences Version 29 (SPSS, version 29) was utilized for data manipulation and other analyses. Analyses were performed only on respondents who completed the entire questionnaire. Statistical significance was determined by setting *p* < 0.05 as the threshold.

Demographic characteristics were categorized and calculated in terms of frequencies and percentages. For the purpose of examining the quality of the items, item discrimination and distribution analysis were conducted to determine interpretability. Item discrimination was evaluated using the upper and lower 27% rule, examining both the difficulty gap between the top and bottom 27% of participants and the correlation coefficient between each item score and the total test score (22).

In order to assess dimensionality and internal consistency reliability, an exploratory factor analysis (EFA) was conducted, followed by a confirmatory factor analysis (CFA) to determine whether the Chinese DLI-R matched the results of the EFA. The full data were randomly split in half for the analyses of EFA (*n* = 555) and CFA (*n* = 592) using SPSS’s built-in function. Pearson correlations were used for the analyses. Considering that most of the factors were only weakly correlated, principal component analysis (PCA) with varimax rotation was adopted. We first extracted factors with eigenvalues > 1 from EFA. It was considered satisfactory to have rotation factor loadings on the primary factor above 0.4 (23). Parallel analysis (PA) was then performed to identify the number of factors using a SPSS syntax script (24), and to compare the results of eigenvalues from PCA. By comparing the raw data eigenvalues with the mean eigenvalues generated from random data, retention continues until the raw data eigenvalue is less than the corresponding mean eigenvalue. The Kaiser–Meyer–Olkin (KMO) measurement and Bartlett’s test of sphericity were used to assess the suitability of the data for PCA. The dataset was considered appropriate for PCA when KMO was over 0.60, and Bartlett’s test of sphericity was statistically significant (*p* < 0.05) (23). The internal consistency reliability was examined using Cronbach’s alpha and McDonald’s omega. While the average of variance extracted (AVE) and composite reliability (CR) were assessed to confirm convergent validity and discriminant ability among subscales, using the formula proposed by Fornell and Larcker (25). Models with AVE > 0.5 and CR > 0.7 were considered adequate (25, 26). Discriminant validity was examined using Pearson’s correlation between DLI-R and CDS.

CFA indicators such as the comparative fit index (CFI), goodness-of-fit index (GFI), non-norm-fitting index (Tucker–Lewis Index, TLI), root mean square error of approximation (RMSEA), and standardized root mean square residual (SRMR) were used to assess the goodness of fit and acceptability of the model. Using the maximum likelihood method, the model was considered to have a reasonable fit if the CFI, GFI, and TLI were more than 0.9, the RMSEA was less than 0.08, and the SRMR was less than 0.09 (27). After confirmation of the measurement model, multigroup confirmatory factor analysis (MGCFA) was conducted to assess the validity of the construct across subgroups. Three levels of measurement invariance (i.e., configural, metric, and scalar). The model was deemed acceptable if the changes in CFI (ΔCFI), TLI (ΔTLI), RMSEA (ΔRMSEA), and SRMR (ΔSRMR) were all less than 0.01 (28). A *p*-value below 0.05 was considered statistically significant.

## 3 Results

### 3.1 Participant characteristics

A total of 1,147 valid responses were received in this study. There was no missing data in the valid responses. A majority of the participants were women (77.5%) and aged 18–34 (46.64%) (ranged 18–74, mean ± SD = 37.31 ± 14.27). Most of them had an education level of college or above (73.6%), were married or cohabited (50.0%), and had siblings (82.7%). However, the majority of them did not have children (50.9%) or hold any religious beliefs (72.3%) (Table 1).

**Table 1:** Socio-demographic characteristics (*n* = 1,147).

Variables		*n*	%
Sex	Male	258	22.5
	Female	889	77.5
Age (year)	18–24	311	27.1
	25–34	224	19.5
	35–44	260	22.7
	45–54	208	18.1
	55–64	81	7.1
	65–74	63	5.5
Education level	Primary school or below	26	2.3
	Secondary school	277	24.1
	College or above	844	73.6
Marital status	Not married	512	44.6
	Married/cohabited	573	50.0
	Separated/divorced	49	4.3
	Widowed	13	1.1
Children	No	584	50.9
	Yes	563	49.1
Sibling(s)	No	198	17.3
	Yes	949	82.7
Religious beliefs	No	829	72.3
	Yes	318	27.7
Employment status	Not employed	413	36.0
	Employed	734	64.0

### 3.2 Exploratory factor analysis

The item discrimination test showed positive discrimination for all the items (Table 2). The KMO was 0.913 in the Chinese DLI-R and 0.837 in the Chinese DLI-9. The Bartlett’s test of sphericity was significant in both Chinese DLI-R ( $\chi_{406}^{2}$ =10247.138; *p* < 0.001) and Chinese DLI-9 ( $\chi_{36}^{2}$ =1255.274; *p* < 0.001), suggesting that the matrices were suitable for factor extraction. For the Chinese DLI-R, a six-factor model was suggested by PCA. However, the result of PA suggested a five-factor solution (Table 3). Then, PCA was restricted to extract 5 factors for further analysis. The items accounted for a cumulative variance of 65.06%. The overall Cronbach’s alpha coefficient was 0.92, while the subscales were 0.78–0.95, with similar results of Omega coefficients, suggesting good internal consistency reliability (Table 4). The correlation between the factors is shown in Table 5. For Chinese DLI-9, a two-factor model was suggested. The items accounted for a cumulative variance of 52.56%. The overall Cronbach’s alpha coefficient was 0.79, while the subscales were 0.82 and 0.56, also suggesting good internal consistency reliability (Tables 4, 6).

**Table 2:** Item analysis of the Chinese DLI-R (*n* = 1,147).

Items	Mean	SD	Skewness	Item discrimination	Cronbach’s *α* if item deleted	Corrected item-total correlation coefficients
1. Talking about death, dying, or grief to a close friend	7.7	2.45	−1.06	−12.83***	0.92	0.37
2. Talking about death, dying, or grief to a child	6.8	2.80	−0.65	−12.89***	0.92	0.37
3. Talking to a grieving person about their loss	5.6	2.99	−0.18	−15.62***	0.92	0.38
4. Talking to a health professional about getting support for a dying person where they live	7.3	2.59	−0.87	−12.98***	0.92	0.35
5. Feed or help a person to eat	8.0	2.21	−1.29	−13.47***	0.92	0.37
6. Bath a person	6.4	3.03	−0.41	−15.99***	0.92	0.41
7. Lift a person or help them move	7.3	2.57	−0.92	−15.07***	0.92	0.40
8. Administer injections	5.6	3.80	−0.17	−17.05***	0.92	0.40
9. Made me more emotionally prepared to support others with death, dying, and bereavement	7.7	2.28	−1.22	−15.17***	0.92	0.44
10. Made me think about what is important and not important in life	8.4	1.91	−1.54	−11.64***	0.92	0.36
11. Developed my wisdom and understanding	8.3	1.90	−1.36	−11.16***	0.92	0.35
12. Made me more compassionate toward myself	8.2	2.00	−1.36	−12.33***	0.92	0.35
13. Made me better prepared to face similar challenges in the future	8.0	2.18	−1.36	−13.71***	0.92	0.40
14. I know the rules and regulations when a person dies at home	5.0	3.08	0.08	−31.33***	0.92	0.63
15. I know what documents are needed when planning for death	4.8	3.10	0.16	−28.33***	0.92	0.61
16. I know enough about the healthcare system to find the support that a dying person needs	5.5	3.15	−0.12	−31.18***	0.92	0.67
17. I know enough to make decisions about funeral services and options	4.8	3.07	0.15	−27.30***	0.92	0.61
18. I know how to access palliative care in my area	5.5	3.17	−0.13	−28.05***	0.92	0.63
19. I know enough about how illnesses progress to make decisions about medical treatments at the end of life	6.5	2.96	−0.68	−23.24***	0.92	0.57
20. I know about the ways that cemetery/ funeral staff can be of help around funerals	6.1	2.98	−0.44	−22.24***	0.92	0.56
21. To get support in the area where I live, for example, from clubs, associations, or volunteer organizations	6.0	3.06	−0.38	−30.87***	0.92	0.69
22. To get help with providing day-to-day care for a person at the end of life	6.2	2.96	−0.53	−30.45***	0.92	0.68
23. To get the equipment that is required for care	5.9	3.06	−0.40	−34.74***	0.92	0.70
24. To get support that is culturally appropriate for a person	5.6	3.08	−0.21	−35.94***	0.92	0.69
25. To get emotional support for myself	6.6	2.93	−0.73	−22.95***	0.92	0.60
26. People with life-threatening illnesses	5.5	3.12	−0.18	−24.28***	0.92	0.59
27. People who are nearing the end of their lives	5.4	3.09	−0.16	−24.00***	0.92	0.59
28. People who are caring for a dying person	5.5	3.08	−0.20	−25.25***	0.92	0.59
29. People who are grieving	5.5	3.09	−0.17	−22.48***	0.92	0.56

*p < 0.05; **p < 0.01; ***p < 0.001.

**Table 3:** Result of parallel analysis (*n* = 555).

Root	Raw Data	Means	Percentile
1	9.64	1.45	1.51
2	3.30	1.38	1.43
3	2.25	1.34	1.37
4	1.91	1.30	1.33
5	1.77	1.26	1.29
6	1.21	1.23	1.26
7	0.83	1.20	1.22
8	0.71	1.17	1.19
9	0.64	1.14	1.16
10	0.62	1.11	1.14
11	0.59	1.08	1.11
12	0.50	1.06	1.08
13	0.48	1.03	1.06
14	0.44	1.01	1.03
15	0.41	0.98	1.01
16	0.39	0.96	0.98
17	0.38	0.94	0.96
18	0.35	0.91	0.94
19	0.33	0.89	0.91
20	0.30	0.87	0.89
21	0.30	0.84	0.87
22	0.27	0.82	0.84
23	0.26	0.80	0.82
24	0.24	0.77	0.80
25	0.22	0.75	0.77
26	0.21	0.72	0.75
27	0.19	0.70	0.72
28	0.14	0.67	0.69
29	0.12	0.63	0.66

**Table 4:** Exploratory factor analysis of the Chinese DLI-R (*n* = 555).

Items	Factor Loading					Communalities
	F1	F2	F3	F4	F5	
17. I know enough to make decisions about funeral services and options	0.75	0.05	0.01	−0.01	0.28	0.65
14. I know the rules and regulations when a person dies at home	0.75	0.09	0.11	0.10	0.21	0.63
15. I know what documents are needed when planning for death	0.74	0.04	0.03	0.05	0.26	0.62
24. To get support that is culturally appropriate for a person	0.74	0.27	0.18	0.12	−0.05	0.67
16. I know enough about the healthcare system to find the support that a dying person needs	0.73	0.12	0.09	0.18	0.14	0.61
23. To get the equipment that is required for care	0.72	0.31	0.13	0.19	−0.08	0.67
21. To get support in the area where I live, for example, from clubs, associations, or volunteer organizations	0.69	0.34	0.20	0.12	−0.03	0.64
18. I know how to access palliative care in my area	0.69	0.21	0.05	0.09	0.15	0.55
22. To get help with providing day-to-day care for a person at the end of life	0.68	0.32	0.24	0.15	−0.06	0.64
19. I know enough about how illnesses progress to make decisions about medical treatments at the end of life	0.65	0.08	0.07	0.23	0.04	0.49
25. To get emotional support for myself	0.61	0.30	0.26	0.14	−0.08	0.55
20. I know about the ways that cemetery/ funeral staff can be of help around funerals	0.61	0.16	0.04	0.07	0.17	0.43
28. People who are caring for a dying person	0.32	0.87	0.03	0.03	0.08	0.87
27. People who are nearing the end of their lives	0.30	0.87	0.03	0.00	0.10	0.86
26. People with life-threatening illnesses	0.30	0.87	0.04	0.01	0.10	0.85
29. People who are grieving	0.25	0.86	0.02	0.06	0.08	0.81
11. Developed my wisdom and understanding	0.12	−0.01	0.85	0.05	0.07	0.74
12. Made me more compassionate toward myself	0.11	0.01	0.80	0.10	0.14	0.69
10. Made me think about what is important and not important in life	0.10	0.07	0.79	0.08	0.12	0.66
13. Made me better prepared to face similar challenges in the future	0.13	0.06	0.78	0.10	0.12	0.65
9. Made me more emotionally prepared to support others with death, dying, and bereavement	0.22	0.03	0.60	0.14	0.31	0.52
5. Feed or help a person to eat	0.10	−0.03	0.11	0.82	0.16	0.72
6. Bath a person	0.19	0.08	0.09	0.81	0.08	0.71
7. Lift a person or help them move	0.16	0.05	0.12	0.80	0.07	0.69
8. Administer injections	0.24	0.03	0.09	0.71	0.02	0.57
2. Talking about death, dying, or grief to a child	0.11	0.06	0.16	0.01	0.79	0.66
1. Talking about death, dying, or grief to a close friend	0.10	0.05	0.15	0.13	0.75	0.61
3. Talking to a grieving person about their loss	0.17	0.08	0.12	−0.01	0.69	0.65
4. Talking to a health professional about getting support for a dying person where they live	0.08	0.06	0.13	0.17	0.68	0.52
Eigenvalues	9.64	3.30	2.25	1.91	1.77	
% of Variance	33.25	11.39	7.75	6.57	6.09	
% of Cumulative Variance	33.25	44.64	52.39	58.96	65.06	
Cronbach’s alpha (α)/ McDonald’s omega (ω)	0.93/0.93	0.95/0.95	0.86/0.87	0.82/0.82	0.78/0.78	0.92/0.92
Average of variance extracted (AVE)	0.49	0.75	0.65	0.62	0.53	
Composite Reliability (CR)	0.92	0.92	0.88	0.87	0.82	

F1, Factual Knowledge and Community knowledge: Accessing help; F2, Community knowledge: Community support group; F3, Experiential Knowledge; F4, Practical knowledge: Hands-on care; F5, Practical knowledge: Talking support.

**Table 5:** Correlation between factors (*n* = 555).

Factors	Practical knowledge: talking support	Practical knowledge: hands-on care	Experiential knowledge	Factual knowledge and community knowledge: accessing help	Community knowledge: community support group
Practical knowledge: Talking support	1				
Practical knowledge: Hands-on care	0.22***	1			
Experiential Knowledge	0.37***	0.29***	1		
Factual Knowledge and Community knowledge: Accessing help	0.30***	0.39***	0.36***	1	
Community knowledge: Community support group	0.20***	0.16***	0.16***	0.55***	1

***p < 0.001.

**Table 6:** Exploratory factor analysis of the Chinese DLI-9 (*n* = 555).

Items	Factor loading		Communalities
	F1	F2	
16. I know enough about the healthcare system to find the support that a dying person needs	0.81	0.37	0.66
18. I know how to access palliative care in my area	0.78	0.30	0.60
24. To get support that is culturally appropriate for a person	0.77	0.35	0.60
15. I know what documents are needed when planning for death	0.77	0.33	0.59
28. People who are caring for a dying person	0.69	0.13	0.49
9. Made me more emotionally prepared to support others with death, dying, and bereavement	0.28	0.80	0.63
13. Made me better prepared to face similar challenges in the future	0.24	0.73	0.53
5. Feed or help a person to eat	0.18	0.58	0.33
3. Talking to a grieving person about their loss	0.32	0.52	0.29
Eigenvalues	3.44	1.29	
% of Variance	38.20	14.36	
% of Cumulative Variance	38.20	52.56	
Cronbach’s alpha (α)/ McDonald’s omega (ω)	0.82/0.82	0.56/0.58	0.79/0.80
Average of variance extracted (AVE)	0.58	0.44	
Composite Reliability (CR)	0.87	0.75	

### 3.3 Confirmatory factor analysis

**Figure 1:**
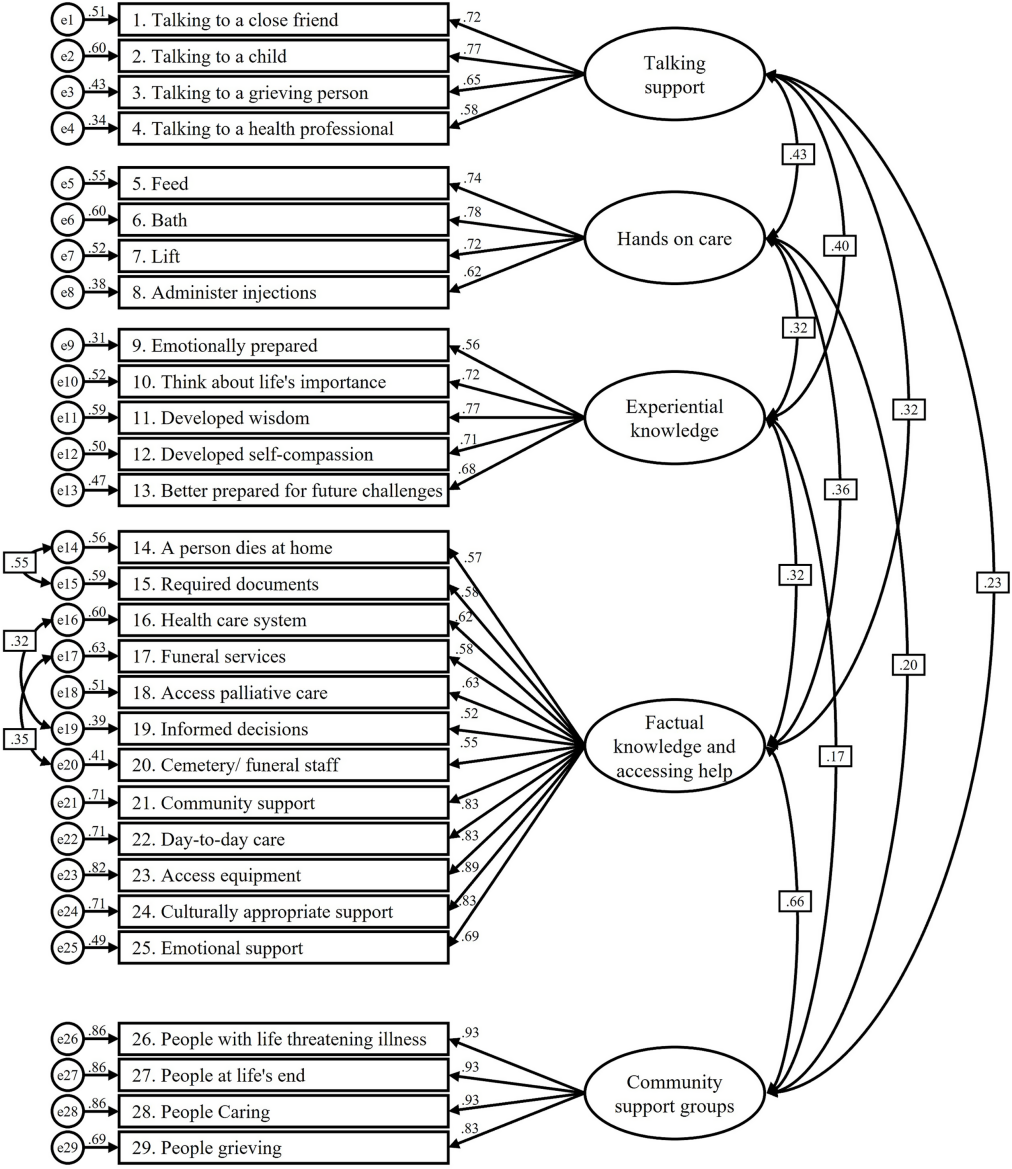
Structural equation model for the fitting model for the Chinese DLI-R.

**Figure 2:**
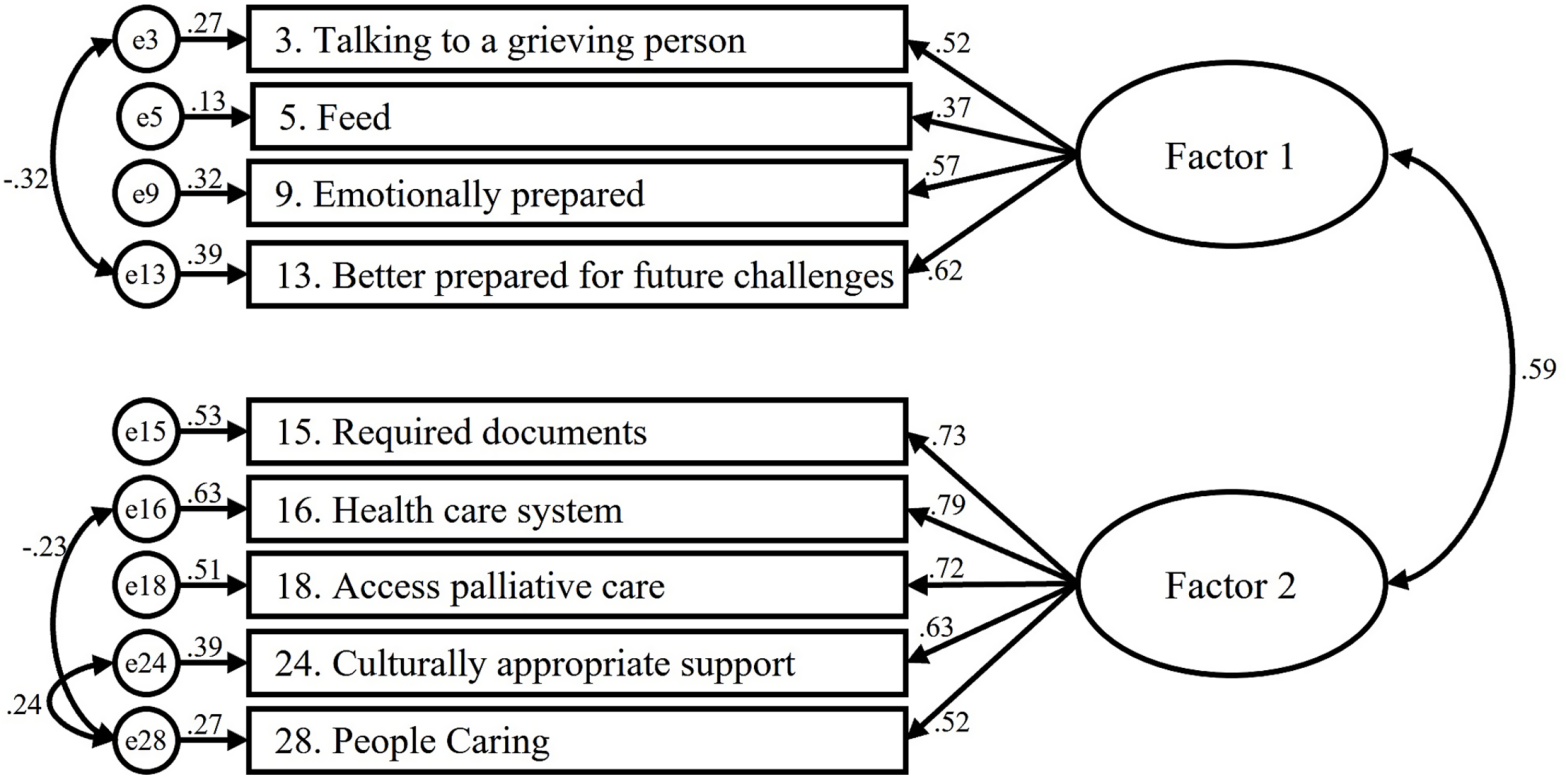
Structural equation model for the fitting model for the Chinese DLI-9.

A sample of 592 was used to conduct CFA. The model fit of CFA for the Chinese DLI-R was as follows: $\chi_{362}^{2}$ =1289.50, CFI = 0.91, GFI = 0.85, TLI = 0.90, RMSEA = 0.07 (90% C.I. = 0.06–0.07), and SRMR = 0.07. In MGCFA, the results showed acceptable fit in the values of CFI, TLI, RMSEA, and SRMR for comparisons of gender, age (18–44 and 45–74 years old), and education level (secondary school or below and college or above) for DLI-R. However, the results of the DLI-9 did not meet optimal standards (Table 7). It was found that all factors had AVE > 0.4 and CR > 0.7, indicating adequate convergent validity and discriminant ability among subscales. The factor loading of the items in CFA ranged from 0.52 to 0.93. The path diagrams for the CFA model are shown in Figures 1, 2.

**Table 7:** Multi-group confirmatory factor analysis for different sub-groups (*n* = 592).

Model	CFI	GFI	TLI	RMSEA (90% CI)	SRMR	Model compare	ΔCFI	ΔTLI	ΔRMSEA	ΔSRMR	Decision
*DLI-R*											
Full sample (*n* = 592)	0.906	0.851	0.895	0.066 (0.062, 0.069)	0.067						
Gender											
M1: Configural invariance	0.899	0.832	0.888	0.039 (0.038, 0.041)	0.067						
M2: Metric invariance	0.900	0.830	0.893	0.038 (0.037, 0.040)	0.067	M1	0.001	0.005	−0.001	0.0002	Accept
M3: Scalar invariance	0.901	0.827	0.901	0.037 (0.035, 0.039)	0.067	M2	0.001	0.008	−0.001	−0.0001	Accept
Age											
M1: Configural invariance	0.899	0.828	0.899	0.037 (0.036, 0.039)	0.067						
M2: Metric invariance	0.899	0.828	0.899	0.037 (0.036, 0.039)	0.067	M1	0.000	0.000	0	0	Accept
M3: Scalar invariance	0.899	0.828	0.900	0.037 (0.036, 0.039)	0.067	M2	0.000	0.001	0	0	Accept
Education level											
M1: Configural invariance	0.893	0.814	0.894	0.038 (0.037, 0.040)	0.067						
M2: Metric invariance	0.893	0.814	0.894	0.038 (0.037, 0.040)	0.067	M1	0	0	−0.005	0	Accept
M3: Scalar invariance	0.893	0.814	0.895	0.038 (0.037, 0.040)	0.067	M2	0	0.001	−0.002	0	Accept
*DLI-9*											
Full sample (*n* = 592)	0.925	0.956	0.897	0.078 (0.065, 0.093)	0.052						
Gender											
M1: Configural invariance	0.925	0.951	0.896	0.045 (0.039, 0.051)	0.052						
M2: Metric invariance	0.928	0.950	0.916	0.041 (0.035, 0.046)	0.052	M1	0.003	0.020	−0.004	0.0003	Reject
M3: Scalar invariance	0.932	0.948	0.937	0.035 (0.030, 0.040)	0.052	M2	0.004	0.021	−0.006	−0.0002	Reject
Age											
M1: Configural invariance	0.925	0.952	0.896	0.046 (0.040, 0.052)	0.052						
M2: Metric invariance	0.923	0.948	0.910	0.043 (0.037, 0.048)	0.052	M1	−0.002	0.014	−0.003	0.0003	Reject
M3: Scalar invariance	0.920	0.943	0.926	0.039 (0.034, 0.044)	0.052	M2	−0.003	0.016	−0.004	−0.0002	Reject
Education level											
M1: Configural invariance	0.910	0.943	0.875	0.050 (0.045, 0.056)	0.052						
M2: Metric invariance	0.912	0.942	0.897	0.046 (0.040, 0.051)	0.051	M1	0.002	0.022	−0.004	−0.0007	Reject
M3: Scalar invariance	0.912	0.937	0.918	0.041 (0.036, 0.046)	0.051	M2	0	0.021	−0.005	0.0005	Reject

The correlation analysis revealed that the total score of the Chinese DLI-R and DLI-9 had a significantly moderate to large correlation with the CDS (Tables 8, 9), demonstrating that the CDS is related to the Chinese DLI-R and DLI-9, but they also measure different aspects.

**Table 8:** Correlation between DLI-R and CDS (*n* = 1,147).

Factors	Chinese DLI-R						CDS								
	F1	F2	F3	F4	F5	Total	A	B	C	D	E	F	G	H	Total
F1. Talking support	1														
F2. Providing hands-on care	0.29***	1													
F3. Experiential knowledge	0.35***	0.29***	1												
F4. Factual knowledge and Others can help me provide end-of-life care	0.32***	0.37***	0.35***	1											
F5. Support groups in my community	0.20***	0.17***	0.16***	0.58***	1										
DLI-R Total	0.54***	0.56***	0.54***	0.91***	0.68***	**1**									
A. Death acceptance	0.35***	0.18***	0.25***	0.43***	0.27***	0.46***	1								
B. Death processing	0.37***	0.37***	0.34***	0.48***	0.28***	0.55***	0.58***	1							
C. Death thinking and expression	0.33***	0.27***	0.40***	0.37***	0.23***	0.45***	0.46***	0.62***	1						
D. Funeral processing	0.32***	0.27***	0.28***	0.60***	0.38***	0.61***	0.50***	0.59***	0.52***	1					
E. Life inspection	0.28***	0.20***	0.24***	0.38***	0.21***	0.41***	0.49***	0.53***	0.54***	0.55***	1				
F. Loss processing	0.45***	0.29***	0.28***	0.49***	0.25***	0.54***	0.56***	0.56***	0.46***	0.59***	0.57***	1			
G. Ability to discuss others’ deaths	0.46***	0.26***	0.29***	0.45***	0.27***	0.52***	0.59***	0.56***	0.48***	0.54***	0.53***	0.72***	1		
H. Ability to discuss own death	0.48***	0.23***	0.28***	0.41***	0.25***	0.48***	0.64***	0.55***	0.52***	0.52***	0.53***	0.67***	0.81***	1	
CDS Total	0.48***	0.33***	0.38***	0.58***	0.34***	0.64***	0.79***	0.80***	0.74***	0.77***	0.73***	0.80***	0.82***	0.82***	**1**

*** *p* < 0.001. Bold indicates the total score of the scale.

**Table 9:** Pearson correlation between DLI and CDS (*n* = 1,147).

Factors	DLI9			CDS								
	F1	F2	Total	A	B	C	D	E	F	G	H	Total
DLI-9: F1	1											
DLI-9: F2	0.421***	1										
DLI-9 Total	0.726***	0.930***	**1**									
A. Death acceptance	0.358***	0.409***	0.456***	1								
B. Death processing	0.452***	0.446***	0.522***	0.583***	1							
C. Death thinking and expression	0.421***	0.330***	0.421***	0.459***	0.621***	1						
D. Funeral processing	0.364***	0.575***	0.584***	0.502***	0.585***	0.517***	1					
E. Life inspection	0.333***	0.348***	0.400***	0.491***	0.527***	0.543***	0.545***	1				
F. Loss processing	0.461***	0.472***	0.545***	0.564***	0.556***	0.455***	0.587***	0.570***	1			
G. Ability to discuss others’ deaths	0.457***	0.443***	0.521***	0.592***	0.559***	0.482***	0.537***	0.529***	0.724***	1		
H. Ability to discuss own death	0.424***	0.394***	0.471***	0.638***	0.553***	0.521***	0.519***	0.525***	0.673***	0.805***	1	
CDS Total	0.519***	0.547***	0.626***	0.791***	0.804***	0.741***	0.768***	0.732***	0.802***	0.817***	0.823***	**1**

***p < 0.001. Bold indicates the total score of the scale.

## 4 Discussion

This study is the first to report the translation and validation of DLI-R and DLI-9 in the Chinese population. As only minor adjustments were made to the scale description and item wording during the development of the DLI-R, these changes were not considered to significantly impact the original DLI, which has been validated and published in various languages (10). To validate a reliable assessment tool for examining the death system from a public health perspective and comparing the effects of relevant policies among diverse cultural contexts, this study translated and performed cultural adaptation to form the Chinese DLI-R, as well as validated the Chinese version of the DLI-9 to ensure that the scale accurately measures the required constructs in the new context. In our exploratory factor analysis of the Chinese version of DLI-R, a five-factor model was suggested instead of the original six factors. Among the four scales of DLI-R, the subscale of Community Knowledge, “Others can help me provide end-of-life care,” was combined with the scale of Factual knowledge to form one larger scale. This may be due to the ambiguous distinction between the items of Factual knowledge, for instance, item 18 “I know how to access palliative care in my area” from the items in Community Knowledge, for instance, I know people who could help me to get support that is culturally appropriate for a person. All these items assessed knowledge of the support that people can access, which was treated as the overall resources available in the system. Similarly, the Swedish DLI validations also found these two factors highly correlated in CFA, and they might be suitable to measure one underlying factor in this instance (5).

The Cronbach’s alpha coefficient of the Chinese DLI-R (0.92) was higher than that of the Chinese DLI-9 (0.79). In the two-factor structure of Chinese DLI-9, Factor 2 indicated a Cronbach’s alpha of 0.56, in which item 3 (Talking to a grieving person about their loss) and item 5 (Feed or help a person to eat) showed the two lowest loadings (0.48 and 0.57). The items in Factor 2 were derived from the subscales of “Practical knowledge” and “Experiential knowledge,” while both items 3 and 5 are from the subscales of “Practical knowledge.” Item 3 also demonstrated a low loading in the previous validation of the Chinese DLI (4). The consistency in these two findings suggests that talking to a grieving person about their loss is not considered practical knowledge among Chinese individuals, as keeping grief private is an implicit norm within Chinese families, guiding individuals to avoid openly expressing grief (29, 30). A study further illustrated this cultural rule by exploring bereaved parents who shared similar experiences of having lost their only child and formed a trustworthy and genuine peer support group. They shared an unspoken consensus that they avoided discussing any topic about their lost children. They saw it as a way to protect each other (31). Recent studies revealed that the Chinese can talk freely about their own death (32); however, they cannot be as free and willing to talk about death to a grieving person (33–35). It is believed that talking about death is still a taboo across the Chinese population. Therefore, talking to a grieving person can comparably be a weak concept in the death system of the Chinese context. Despite this, the loading of item 3 could achieve the threshold of 0.4. Considering that talking to a grieving person is so essential in reflecting one’s or community’s death literacy in literature (11), the item was counted as theoretically adequate for the construct of Chinese DLI-9.

Despite the AVEs of Factual Knowledge in the DLI-R and factor 2 in the DLI-9 being lower than the recommended 0.5, the internal consistency reliability of the scale (Cronbach’s Alpha and McDonald’s Omega) and discriminant ability (composite reliability) are satisfactory. Furthermore, it is considered more conservative to estimate the validity of a measurement model by using the AVE (25), and the authors of the DLI-R no longer recommend using the DLI-9 factors alone. Therefore, the scale is still a reliable measurement tool. Although known-groups validity was not performed in the study, AVE and CR were assessed as the indicators of construct validity, which assesses whether a test measures the intended construct. Both AVE and CR adequately indicated that the factors were well correlated with the latent variable (AVE > 0.5 and CR > 0.7). Moreover, the significant correlation between factors of DLI and CDS demonstrated strong discriminant validity.

Regarding feeding or helping a person to eat as an example of practice knowledge in death literacy, its factor loading in the Chinese DLI-R was higher than in the Chinese DLI-9, as was the case in the Chinese DLI, as reported by Che et al. (4). Since the KMO value indicated the factoring was suitable, its low loading in the Chinese DLI-9 might reflect its unstable implication in death literacy. Bellamy and Gott’s study (36) reported that Chinese families tended to provide “hands-on” care to their older family members. However, Chen (37) found that there was a transition of filial responsibilities, filial behavior, and intergenerational relationships. In a previous filial piety study, over 70% of residents in Macao, where the majority of these study participants came from, were found to adopt a reciprocal rather than a traditional authoritarian approach in their filial piety representation in a family (38). As the authoritarian belief was fading away, Chinese families might value reciprocal affections rather than “hands-on” care (37). In such circumstances, it is recommended not to use DLI-9 as a single scale in the Chinese context.

Literature emphasized that the developing process of the short-form scale should combine both qualitative content and statistical psychometric analysis (39). As reported by the research team of the original DLI-9, which was not developed from literature and rigorous qualitative research, discriminant validity was examined by conducting the correlations between the overall score and factors of the Chinese DLI-R and DLI-9 and Bugen’s Coping with Death Scale. Our study indicated that the total and subscale scores of both Chinese DLI-R and DLI-9 were significantly correlated with the total and all subscale scores of Bugen’s Coping with Death Scale. The finding of Chinese DLI-R was similar to the correlation found between DLI and Bugen’s Coping with Death Scale (1), while Chinese DLI-9 demonstrated a strong correlation with Bugen’s Coping with Death Scale. It implied that DLI-9 or only Chinese DLI-9 might not be sensitive enough to differentiate from the measures from Bugen’s Coping with Death Scale, which was developed with psychometric properties to predict individuals who engage in preparing for death (20). Our CFA analysis also indicated measurement invariance in gender and education level. Higher variance was found in the male group and secondary or lower education group in DLI-9 than DLI-R; a potential discrepancy may be possible when DLI-9 is used in men and the population with lower education. Compared to the DLI-R, the discriminative ability of its short form, the DLI-9, might be limited, possibly in revealing one’s community knowledge of the death system. Taken together, since DLI-9 was developed to be used in community and practical settings where ease of completion is paramount, it showed significant limitations in application for evaluating studies, in a male majority or low education population in the Chinese context.

This study has several limitations that may affect the interpretation and application of the results. The participants resided in three cities in the southern part of China, where they shared different economic and cultural characteristics. The results of validated Chinese DLI-R and DLI-9 may be able to generalized to Chinese people who live in cities possessing similar characteristics. However, they may underrepresent the Chinese population of Northern China or those who live in cities that do not possess the characteristics of these three cities. Even so, the sample size is satisfactory, and the validated tool was translated into both traditional and simplified Chinese and can be read by all Chinese speakers. Due to the exclusive use of self-report instruments, lack of reverse-coded items, and test–retest reliability, multiple procedures were implemented to address potential issues arising from common method bias and acquiescence bias. First, participants were assured anonymity and confidentiality and instructed to answer honestly. Survey items were clearly worded to prevent ambiguity or bias. Second, to address acquiescence bias, the scale design incorporated varied item formats and balanced response options to discourage uniform agreement. Implementing these procedures may minimize potential biases and strengthen the credibility of the results.

Since convenience and snowball sampling were adopted, the distribution of demographic background was neither stratified nor randomized. The sample appeared to have more women, highly educated, and medical professionals, which may introduce biases. Therefore, there is a limitation in generalizing the results to the population in the region. For example, gender bias can lead to findings that overlook the experiences and viewpoints of men or non-binary people. Moreover, the gender differences observed in model fit may reflect that men and women interpret or respond to the measurement items differently. It is also possible that the relatively small male sample size (*n* = 132) used in the CFA may affect the stability and reliability of the results due to insufficient sample size. It is recommended to interpret the results for the male sample with caution and to investigate the reasons for these differences in future research in order to better understand the specific challenges or needs faced in assessing death literacy among men. Similarly, educational bias may cause results to be more representative of individuals with higher education and the cognitive resources available and accessible to them. Therefore, it is important to interpret these findings with caution, as they may not accurately reflect the broader diversity of the general population. Ensuring more inclusive and representative sampling in future studies will improve the validity and applicability of research outcomes, allowing findings to more effectively address the needs of the broader population.

## 5 Conclusion

This study is the first to report the translation and validation of the DLI-R and its short form, the DLI-9, among the southern Chinese population. A five-factor construct was suggested for the Chinese DLI-R for assessing death literacy among Chinese individuals with good validity and reliability, while some cultural adaptation and demographic considerations were raised for the Chinese DLI-9. The Chinese DLI-9 was recommended not to be used as a single scale in the Chinese context, and further research is needed to learn about its performance in evaluating studies in a different cultural context.

## Data Availability

The raw data supporting the conclusions of this article will be made available by the authors, without undue reservation.
